# Camrelizumab Plus Apatinib in Patients With Recurrent or Metastatic Nasopharyngeal Carcinoma: An Open-Label, Single-Arm, Phase II Study

**DOI:** 10.1200/JCO.22.01450

**Published:** 2023-02-03

**Authors:** Xi Ding, Wei-Jing Zhang, Rui You, Xiong Zou, Zhi-Qiang Wang, Yan-Feng Ouyang, Lan Peng, You-Ping Liu, Chong-Yang Duan, Qi Yang, Chao Lin, Yu-Long Xie, Si-Yuan Chen, Yong-Long Liu, Chen-Mei Gu, Ruo-Qi Xie, Pei-Yu Huang, Ming-Huang Hong, Yi-Jun Hua, Ming-Yuan Chen

**Affiliations:** ^1^ Department of Nasopharyngeal Carcinoma, Sun Yat-sen University Cancer Center, Guangzhou, P.R. China; ^2^ Sun Yat-sen University Cancer Center, State Key Laboratory of Oncology in South China, Collaborative Innovation Center for Cancer Medicine, Guangzhou, P.R. China; ^3^ Guangdong Key Laboratory of Nasopharyngeal Carcinoma Diagnosis and Therapy, Guangzhou, P.R. China; ^4^ Department of Medical Imaging, Sun Yat-sen University Cancer Center, Guangzhou, P.R. China; ^5^ Department of Radiation Oncology, First Affiliated Hospital of Kunming Medical University, Kunming, P.R. China; ^6^ Department of Biostatistics, School of Public Health, Southern Medical University, Guangzhou, P.R. China; ^7^ Department of Clinical Research, Sun Yat-sen University Cancer Center, Guangzhou, P.R. China

## Abstract

Immune checkpoint inhibitors combined with antiangiogenic therapy reportedly have potential synergistic antitumor activity. We investigated the activity and safety of this regimen for recurrent/metastatic nasopharyngeal carcinoma (NPC).

**METHODS:**

This single-arm, Simon two-stage study enrolled patients with recurrent/metastatic NPC who were refractory to at least first-line systemic therapy and treatment-naive to immune checkpoint inhibitors. The patients received camrelizumab 200 mg once every 3 weeks and apatinib 250 mg once per day. The primary end point was the objective response rate. Key secondary end points included disease control rate, progression-free survival, duration of response, overall survival, and safety.

**RESULTS:**

Between October 14, 2020, and December 23, 2021, 58 patients were enrolled, and all were included in the efficacy and safety analysis set. The objective response rate was 65.5% (95% CI, 51.9 to 77.5), and the disease control rate was 86.2% (95% CI, 74.6 to 93.9). The median duration of response was not reached, and the median progression-free survival was 10.4 months (95% CI, 7.2 to 13.6), with a median follow-up duration of 12.4 months (range, 2.1-19.9 months). Treatment-related adverse events (TRAEs) of grade 3 or higher were reported in 34 (58.6%) patients, with the most common being hypertension (19.0%), nasopharyngeal necrosis (15.5%), headache (12.1%), AST elevation (10.3%), and creatine phosphokinase elevation (10.3%). Sixteen (27.6%) patients discontinued apatinib treatment before progression because of unbearable TRAEs, and the most common complication was nasopharyngeal necrosis (9/16; 56.3%). Recurrent nasopharyngeal lesions (odds ratio, 5.94 [95% CI, 1.45 to 24.24]) and reirradiation (odds ratio, 5.33 [95% CI, 1.15 to 24.79]) were significantly positively correlated with nasopharyngeal necrosis.

**CONCLUSION:**

Camrelizumab plus apatinib had promising antitumor activity in patients with refractory recurrent/metastatic NPC who failed first-line therapy. Moderate to severe TRAEs were experienced by 58.6%, including nasopharyngeal necrosis associated with local recurrence and a history of reirradiation.

## INTRODUCTION

Nasopharyngeal carcinoma (NPC) has the highest prevalence in Southeastern Asia, with age-standardized rates being 22.2-27.2 per 100,000 among males.^1^ The incidence of locoregional recurrence or distant metastases in NPC endemic areas is approximately 20%.^2,3^

**CONTEXT**

**Key Objective**
There remains an unmet clinical need for the promising posterior line treatment for refractory nasopharyngeal carcinoma (NPC). Programmed cell death (ligand)–1 inhibitors combined with antiangiogenic therapy reportedly have potential synergistic antitumor activity. To our knowledge, this single-arm, Simon two-stage trial is the first trial to demonstrate the activity and safety of camrelizumab (an anti–PD-1 antibody) plus apatinib (a tyrosine kinase inhibitor targeting VEGFR2) in patients with recurrent/metastatic NPC who were refractory to at least one line of platinum-containing systemic therapy.
**Knowledge Generated**
Camrelizumab plus apatinib had promising antitumor activity: objective response rate was 65.5% and disease control rate was 86.2%; median duration of response was not reached and median progression-free survival was 10.4 months (median follow-up, 12.4 months). 58.6% of patients experienced grade ≥ 3 treatment-related adverse events (TRAEs), and most of them could be resolved by dose interruption or reduction. One of the TRAEs associated with apatinib, nasopharyngeal necrosis, requires attention, and nasopharyngeal recurrence and reirradiation may be risk factors for it.
**Relevance *(M.L. Gillison)***
In patients with platinum refractory, recurrent or metastatic, endemic NPC, the combination of a PD-1 immune checkpoint inhibitor (ie, camrelizumab) and an oral VEGFR2 inhibitor (ie, apatinib) demonstrated remarkable clinical activity. A new safety signal of moderate to severe nasopharyngeal necrosis associated with bleeding was identified with this combination. Active tumor in the nasopharynx and a history of reirradiation were associated with this TRAE.*
  *Relevance section written by *JCO* Associate Editor Maura L. Gillison, MD, PhD.

The gemcitabine plus cisplatin regimen is the standard-of-care first-line treatment for patients with metastatic or recurrent NPC unsuitable for local treatment.^4^ The addition of a programmed cell death protein-1 (PD-1) inhibitor further improved the efficacy, with a median progression-free survival (PFS) of 9.6-11.7 months (an updated figure is 21.4),^5-8^ and has become a new first-line recommended regimen. However, patients who are refractory to first-line therapy have no strong recommended regimen and few effective options.

Several PD-1 inhibitors have been approved by the US Food and Drug Administration for the subsequent-line treatment of recurrent or metastatic NPC (RM-NPC). Although a modest response rate (20.5%-34.0%) was recorded with PD-1 inhibitor monotherapy, the responses persisted (median > 12 months).^9-12^ Considering the long-term efficacy and low toxicity, synergistic combinations of PD-1 inhibitors with other therapies have emerged as promising strategies.

Angiogenesis is a validated target in NPC.^13-15^ Moreover, several preclinical studies indicated that antiangiogenic therapy improved the efficacy of immunotherapy,^16^ which has been validated by several clinical trials and approved by the US Food and Drug Administration for treating endometrial carcinoma,^17^ hepatocellular carcinoma,^18^ renal carcinoma,^19-21^ etc.

Camrelizumab is a fully humanized, high-affinity monoclonal antibody that binds to PD-1. Apatinib selectively inhibits vascular endothelial growth factor receptor-2 (VEGFR-2)^22^ and showed activity against RM-NPC in several reports.^23-25^ In this phase II study, we assessed the antitumor activity and safety of camrelizumab plus apatinib in patients with NPC who were refractory to one line of systemic therapy.

## METHODS

### Study Design and Participants

This is an open-label, single-arm, phase II trial to assess the preliminary antitumor activity and safety of camrelizumab plus apatinib for patients with RM-NPC. The trial was approved by the Ethics Committee of the Sun Yat-sen University Cancer Center and registered on ClinicalTrials.gov (identifier: NCT04586088). All participants provided written informed consents.

Patients with histologically confirmed NPC who were refractory to at least one line of systemic chemotherapy, or had disease progression within 6 months after induction, concurrent, or adjuvant chemoradiotherapy were enrolled. Other major eligibility criteria included age 18-70 years, recurrent NPC unsuitable for radiotherapy and surgery, an Eastern Cooperative Oncology Group performance status score of 0 or 1, and at least one measurable lesion. The main exclusion criteria included prior therapy with immunotherapy or any agents targeting VEGF(R), any factors affecting oral drug absorption, any grade ≥ 2 hemorrhages within 4 weeks, and tumor invasion to major vessels or obvious nasopharyngeal necrosis.^26,27^ Full eligibility criteria are listed in the Protocol (online only).

### Study Treatment

Apatinib 250 mg was administered orally once daily and camrelizumab 200 mg was administered intravenously once every 3 weeks, until disease progression, death, unacceptable toxicities, or at the patient's request.

Dose modifications of camrelizumab were not permitted. Dose interruptions and reductions (1-2 times) of apatinib were permitted for toxicities that were not relieved by supportive care, with a first dose reduction to 250 mg for 5 days on-2 days off and a second reduction to 250 mg every other day. In cases of high-risk major bleeding (such as nasopharyngeal necrosis invading the internal carotid artery [ICA]) assessed by the investigator, apatinib administration was discontinued. Details of dose modifications and supportive measures are provided in the protocol.

### Assessments

Responses were assessed by independent radiologists according to RECIST v1.1, every three cycles in the first 6 months and every 3 months thereafter. Safety evaluations were examined in every cycle by investigators. Adverse event (AE) data were collected up to 30 days after treatment discontinuation, immune-related AE (irAE) data were collected up to 90 days after the last dose of camrelizumab, and severity was graded using the National Cancer Institute-Common Terminology Criteria for Adverse Events v5.0.

The immunohistochemical analysis of programmed cell death ligand-1 (PD-L1; antibody: CST13684)^28^ expression was conducted and evaluated by certified pathologists. PD-L1 protein expression was determined using the combined positive score (CPS), defined as the number of PD-L1–stained cells divided by the total number of viable tumor cells and multiplied by 100. We set a range of thresholds for CPS (≥ 1, 10, or 25).^5,9-11^

Whole-exome sequencing (Illumina sequencing platform) was performed to calculate the tumor mutational burden (TMB) score, defined as the number of somatic nonsynonymous mutations per megabase.^29^

### End Points

The primary end point was the objective response rate (ORR), defined as the proportion of patients with complete response (CR) or partial response (PR) according to RECIST v1.1. The secondary end points included PFS, defined as the time from treatment initiation to disease progression or death from any cause; overall survival (OS), defined as the survival time until death from any cause; duration of response (DoR), defined as the time from the first evidence of response to disease progression or death; disease control rate (DCR), defined as the proportion of patients who achieved CR, PR, or stable disease; and safety evaluation.

Efficacy analysis was performed in the intention-to-treat (ITT) population. Safety analyses were performed on all patients who received at least one dose of study treatment.

### Statistical Analysis

The statistical design was based on Simon's two-stage phase II optimal design (power of 80% and one-sided α of .05) to rule out an ORR of 25% and a target ORR of 43%. If there were ≥ 6 responders within the first 18 patients, the cohort would expand to 51 patients and the outcome would be positive if more than 17 patients achieved responses. Considering a 10% dropout rate, a total of 57 patients were needed.

The ORR and 95% CI were calculated using the Clopper-Pearson method and compared between groups by using Fisher's exact test or chi-square test, as appropriate. The DoR, PFS, and OS were plotted using the Kaplan-Meier method. The log-rank test was used to compare Kaplan-Meier curves, and Cox proportional hazards model was adopted to determine the hazard ratio (HR) and its associated 95% CI and *P* value. The association of clinical characteristics with nasopharyngeal necrosis was estimated using the odds ratio (OR) and 95% CI via logistic regression. Other clinical outcomes, demographic characteristics, and safety were summarized descriptively. We performed all statistical tests using SPSS v25.0.

## RESULTS

**FIG 1.**
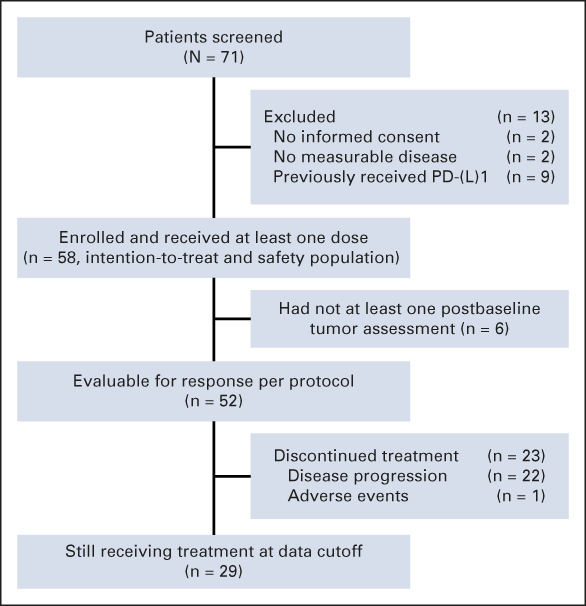
Trial profile. PD-(L)1, programmed cell death (ligand)-1.

**FIG 2.**
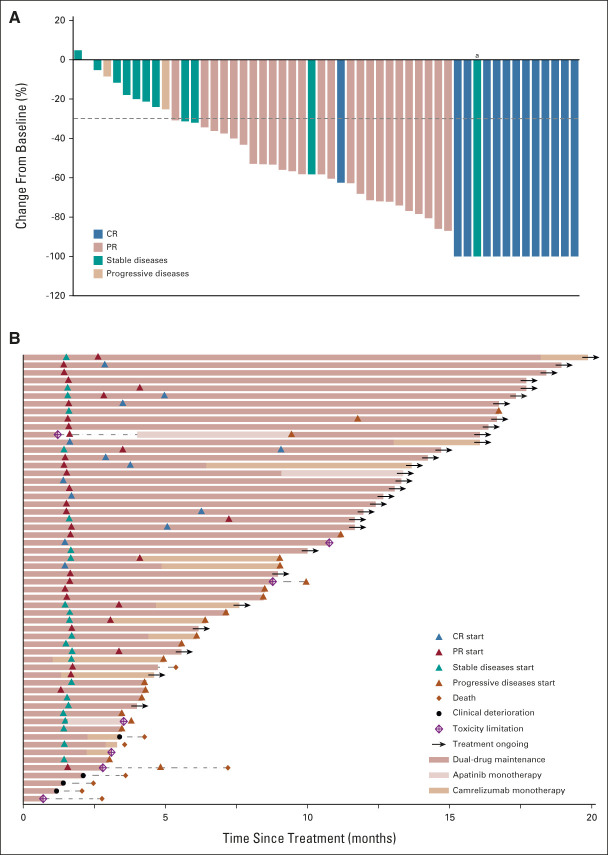
Antitumor activity. The patients in the efficacy-evaluable population were included (n = 52). (A) Best percentage change from baseline in the target lesion. The dashed line at −30% change represents the RECIST version 1.1 cutoff to define PR or CR. (B) Treatment exposure and response duration. ^a^The target lesions of this patient achieved CR at C3D1, but he died at the end of the fifth course, and no imaging was provided for confirmation. CR, complete response; PR, partial response.

Between October 14, 2020, and December 23, 2021, 71 patients were assessed for eligibility, of whom 58 patients were enrolled and received at least one dose of the study regimen (ITT and safety set; Fig 1). Around the first scheduled postbaseline tumor assessment, six patients terminated the treatment without any imaging available for review: one refused the regimen because of dyspepsia and malnutrition; one delayed the re-examination because of grade 4 liver dysfunction and died before the next appointment; one could not undergo examination because of the local COVID-19 outbreak; and three had clinical deterioration deemed unrelated to the study regimen and died in 4 months. Therefore, treatment responses were evaluable only for 52 patients (Fig 2).

Twenty-five patients (43.1%) had locoregional recurrence only, 18 (31.0%) had distant metastases only, and 15 patients (25.9%) had both locoregional recurrence and metastases. All patients received at least one line of prior systemic therapy, with 10 (17.2%) receiving ≥ 2 lines. The baseline characteristics of the population are summarized in Table 1 and the Data Supplement (online only).

**TABLE 1.**
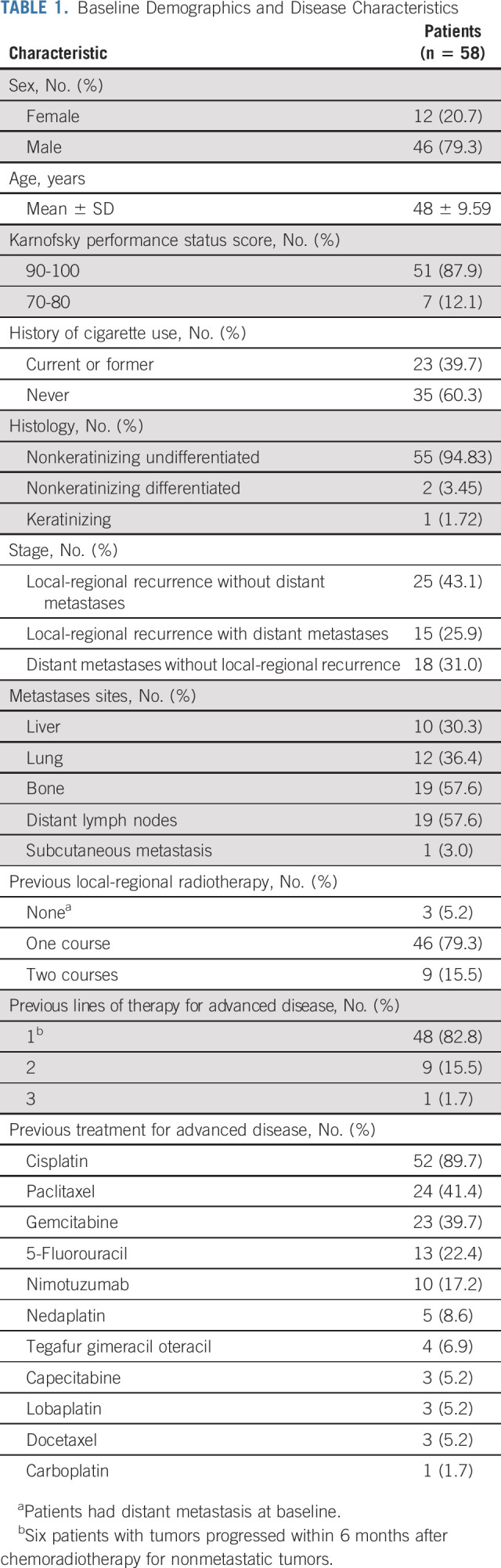
Baseline Demographics and Disease Characteristics

As of the data cutoff (June 2, 2022), the median follow-up was 12.4 months (range, 2.1-19.9 months). Twenty-nine patients (48.3%) discontinued the study treatment, with 26 developing disease progression and three experiencing unacceptable AEs (two refusals and one ketoacidosis). Two patients with disease progression rechallenged the study regimen (Data Supplement).

### Antitumor Activity

Of the first 18 patients enrolled, confirmed responses were noted in 12 patients, and the trial continued to full accrual.

**FIG 3.**
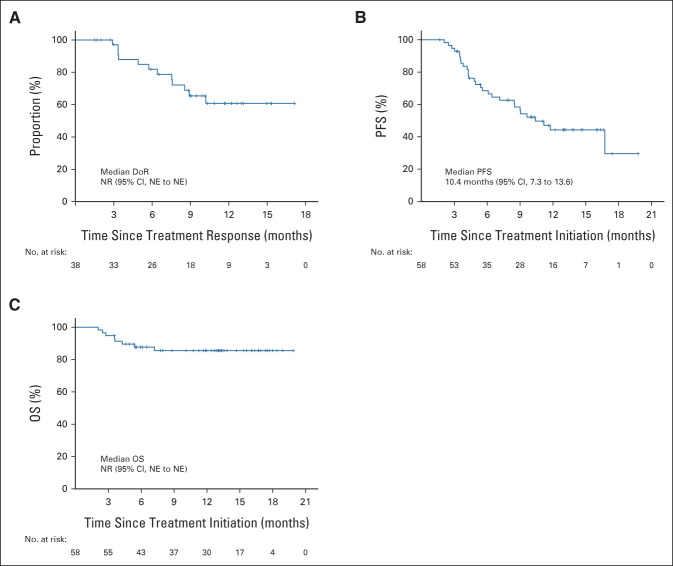
Kaplan-Meier curves of the DoR, PFS, and OS. (A) The DoR was assessed in responders (n = 38). (B) PFS and (C) OS were assessed in the intention-to-treat population (n = 58). DoR, duration of response; NE, not evaluable; NR, not reached; OS, overall survival; PFS, progression-free survival.

In the ITT set, 38 patients (65.5% [95% CI, 51.9 to 77.5]) achieved a confirmed objective response, with 13 CRs (22.4%) and 25 PRs (43.1%; Table 2; Data Supplement). The DCR was 86.2% (95% CI, 74.6 to 93.9). Fifty (96.2%) of the 52 response-evaluable patients had tumor shrinkage in their target lesions (Fig 2A). The median time to achieve a response was 2.1 months (interquartile range, 1.4-3.1 months; Fig 2B) and the median DoR was not reached (Fig 3A). Twenty-six of the 38 responders' (68.4% [95% CI, 51.4 to 82.5]) responses lasted at least 6 months, while nine of them lasted ≥ 12 months; and 25 responses (65.8% [95% CI, 48.7 to 80.4]) were ongoing. The median PFS was 10.4 months (95% CI, 7.2 to 13.6) and the PFS rate at 12 months was 44.3% (95% CI, 29.5 to 58.6). Eight (13.8%) deaths occurred and the median OS was not reached, with an 18-month OS rate of 85.7% (95% CI, 76.2 to 95.0; Fig 3).

**TABLE 2.**
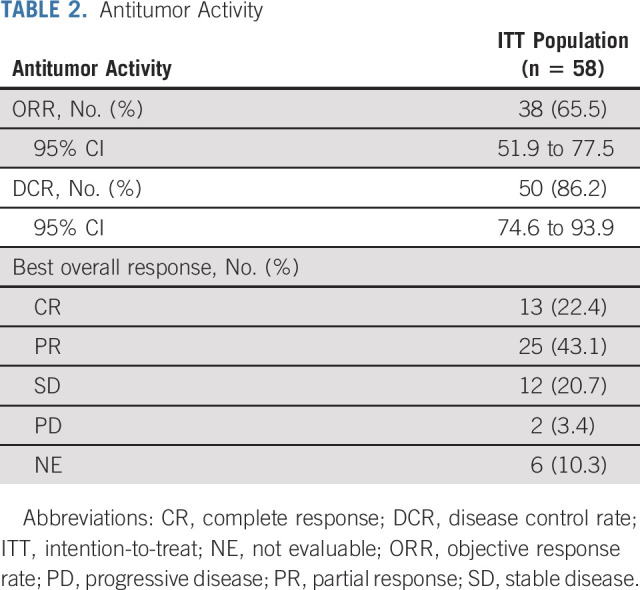
Antitumor Activity

Patients who had locoregional recurrence only exhibited an ORR of 80.0%, while that for patients with metastatic lesions was 54.5% (*P* = .043). Instead, the DoR for the metastatic patients showed a nonsignificantly better trend (HR, 0.54 [95% CI, 0.16 to 1.78]; *P* = .308), and the PFS of the two groups was similar (HR, 1.11 [95% CI, 0.53 to 2.33]; *P* = .784; Data Supplement).

### Safety

All patients experienced at least one treatment-related AE (TRAE). Grade 3-4 TRAEs were observed in 34 patients (58.6%), and the most common TRAEs were hypertension (19.0%), nasopharyngeal necrosis (15.5%), headache (12.1%), AST elevation (10.3%), and creatine phosphokinase elevation (10.3%; Table 3). irAEs of any grade occurred in 42 (72.4%) patients. Most irAEs were grade 1-2 (n = 40; 69.0%), two patients (3.4%) had grade 3 rash or fever, and another two (3.4%) had grade 4 pneumonia or hyperglycemia (Table 3). No treatment-related deaths occurred.

**TABLE 3.**
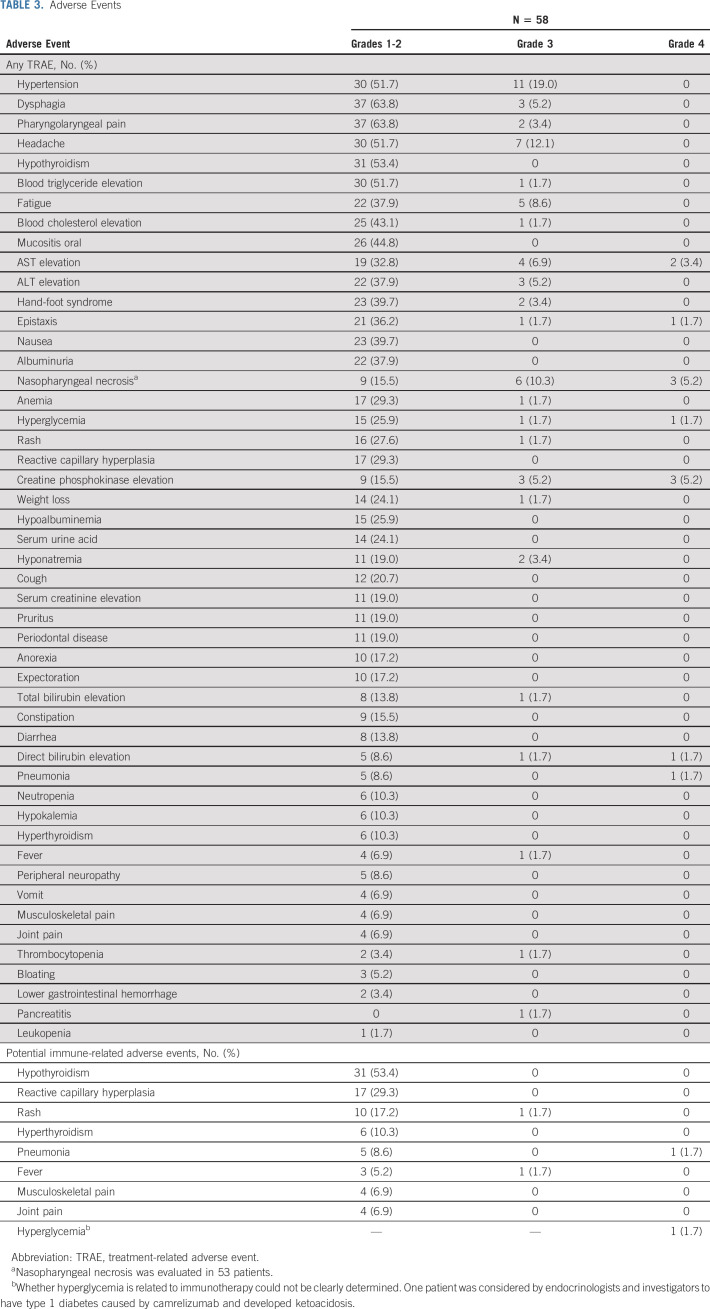
Adverse Events

Fifty-one patients (87.9%) required one or more interruptions for apatinib, and dose reductions were observed in 26 patients (44.8%). The most common reasons for apatinib dose reductions were headache (n = 11; 19.0%) and liver dysfunction (n = 7; 12.1%). Owing to AEs, 16 patients terminated apatinib ahead of progression: 11 complied with medical advice because of grade 3-4 nasopharyngeal necrosis (n = 9; 56.3%), clinical worsening (n = 1; 6.3%), or debridement for necrosis (n = 1; 6.3%); three paused apatinib because of grade 3-4 liver dysfunction (n = 2; 12.5%) or ketoacidosis (n = 1; 6.3%) and then progressed before restarting; and two refused further administration, although the AEs were manageable (grade 2 epistaxis plus grade 3 hand-foot syndrome and fatigue plus dyspepsia).

Five patients did not undergo a post-treatment endoscopic evaluation, and the assessment of nasopharyngeal necrosis was available in 53 patients, with 18 developing it. The median time to the occurrence of necrosis was 2.3 months (range, 0.7-9.2 months). The correlation test revealed a significant positive correlation between necrosis and recurrent nasopharyngeal lesions (OR, 5.94 [95% CI, 1.45 to 24.24]; especially recurrent T3-4) or nasopharyngeal reirradiation (OR, 5.33 [95% CI, 1.15 to 24.79]; Data Supplement). Among the 18 patients with nasopharyngeal necrosis, one underwent ICA embolization when the necrotic foci invaded (grade 4 necrosis, Data Supplement); two had necrosis devouring the ICAs, with ICA occlusion spontaneously (grade 4 necrosis, Data Supplement) or embolization before enrollment; six had grade 1-2 epistaxis; and two developed grade 3-4 epistaxis attributed to necrosis invading the internal jugular veins (conjectured) or the descending palatine artery, which were cured by nasal packing or vascular embolization (Data Supplement).

### Post Hoc Analysis

The ORRs were 76.7% for patients with dose reduction and/or permanent discontinuation (n = 30) and 53.6% for patients with sustained medication without reduction (dose pause permitted, n = 28); however, the difference of ORR between the two subgroups was not significant (*P* = .064). The DoR (HR, 2.02 [95% CI, 0.55 to 7.46]; *P* = .292) and PFS (HR, 1.03 [95% CI, 0.49 to 2.16], *P* = .944) were indistinguishable between the two groups (Data Supplement).

Using CPS ≥ 10 as a cutoff, no difference in ORR between the PD-L1–positive patients (n = 32) and PD-L1–negative ones (n = 15) was observed (65.6% *v* 66.7%, *P* = .944). However, positive patients had a significantly longer DoR than others (HR, 0.20 [95% CI, 0.05 to 0.73], *P* = .015), and the median PFS was 16.7 months in the positive group and 9.0 months in the negative one (HR, 0.50 [95% CI, 0.22 to 1.13], *P* = .095; Data Supplement). Similar results were obtained when the threshold was set to 25, although the difference in the DoR was nonsignificant (HR, 0.30 [95% CI, 0.08 to 1.16], *P* = .080). When using CPS ≥ 1 as the cutoff, only six patients had negative tumors, and none of the efficacy outcomes showed significant differences between the two groups (Data Supplement).

TMB information was available for 23 patients (39.7%), and they were classified into the low- and high-TMB subgroups using the median TMB value (1.26 muts/Mb). No significant differences in ORR, PFS, or DoR were observed between the two groups (Data Supplement).

## DISCUSSION

To our knowledge, this is the first prospective study to evaluate the antitumor activity and safety of immunotherapy combined with antiangiogenesis therapy for RM-NPC. Our results revealed that camrelizumab plus apatinib showed promising antitumor activity and a manageable toxicity profile. Nasopharyngeal necrosis was the most frequent and important cause of apatinib discontinuation and was significantly associated with nasopharyngeal recurrence and reirradiation.

There are limited effective options for patients with RM-NPC who suffer from progression after first-line chemotherapy. Single-agent chemotherapy, considered a standard subsequent-line treatment, exhibited an ORR of 23.3%, with a median PFS of 5.5 months.^30^ Similarly, immune checkpoint inhibitors demonstrated modest antitumor efficacy, with ORRs ranging from 20.5% to 34% and a median PFS of 1.9-6.5 months.^9-12^ Apatinib monotherapy also exhibited a comparable ORR (31.4%-36.4%) and median PFS (3.9-9 months).^23,31,32^ However, when patients were treated with apatinib plus an immune checkpoint inhibitor, the efficacy was boosted, with an ORR of 65.5%, a DCR of 86.2%, and a median PFS of 10.4 months in this study. Increasing evidence has demonstrated that the appropriate administration of antiangiogenic agents (including apatinib)^33^ can normalize tumor vascular network, which could (in) directly alleviate hypoxia, promote T-cell infiltration, induce M1 macrophage polarization,^34^ decrease the recruitment of Treg and myeloid suppressor cell,^35^ and downregulate inhibitory immune checkpoints such as PD-L1,^33^ thereby converting the tumor immune environment from immune-suppressive to immune-supportive.^16,36^ In addition, immunotherapy was also shown to promote tumor vascular normalization.^37^ These pieces of evidence indicate that combining antiangiogenic agents with immunotherapy improves efficacy by creating positive feedback loops that reinforce each other.^33,38,39^ This combination has been verified to be promising in many other solid tumors.^17-21^ Moreover, the efficacy of single-agent apatinib or apatinib plus camrelizumab was similar to that of other VEGFR-tyrosine kinase inhibitors (TKIs) or combined with PD-(L)1 inhibitors, such as lenvatinib/regorafenib/cabozantinib combined with pembrolizumab/nivolumab/atezolizumab (Data Supplement). This suggests that the impressive efficacy of apatinib plus camrelizumab may also be achieved by other antiangiogenic TKIs and PD-(L)1 inhibitors.

The safety profiles of camrelizumab and apatinib in NPC were consistent with those in other solid tumors^40-44^ or the same type of medication (Data Supplement). Moreover, AEs for either drug alone coincided with the respective instructions. Hypertension (70.7%) was the most frequent AE attributed to apatinib, and hypothyroidism (58.6%) was the most common irAE, which was similar to that in previous studies, with incidences of 57.6%-84.4%^41,42,45^ and 30.8%-46.0%,^5,6^ respectively. Some AEs, such as hepatic toxicities, fatigue, and gastrointestinal symptoms, were probably because of the combination therapy, and most of them could be resolved by apatinib dose adjustment, which suggested that these AEs were more likely caused by apatinib than by camrelizumab. The incidences of dysphagia, pharyngolaryngeal pain, and headache in this trial were higher than those observed within other solid tumors, which may be due to the difference in primary tumor types.

In the current study, nasopharyngeal necrosis and its associated severe epistaxis were key limiting factors for the long-term maintenance of apatinib. Two patients developed grade 4 necrosis with a high risk of lethal ICA rupture. Such deadly incidents have been previously reported when using anti-VEGFR TKIs in treating NPC.^15,46^ Therefore, it is necessary to screen out patients who harbor a high possibility of post-treatment necrosis. We found that reirradiation and recurrent nasopharyngeal lesions (especially rT3-4) were high-risk factors for nasopharyngeal necrosis, which was partially consistent with our previous finding.^27^ Remarkably, ICA embolization was useful in our clinical practice to prevent ICA rupture when detecting the approaching necrotic foci, and none of the patients died due to lethal bleeding in this study. Therefore, we suggest that physicians should vigilantly identify patients with a high probability of necrosis before formulating treatment plans, detect necrosis as early as possible through regular endoscopy or magnetic resonance imaging,^26^ and provide timely intervention measures, such as ICA embolization or stent implantation, to avoid fatal massive hemorrhage.^47^

Moreover, we suppose that because nasopharyngeal necrosis was associated with local recurrence and limited the long-term use of apatinib, an ORR advantage for patients with locoregional recurrence could not reflect in the PFS. This is based on our results showing that patients who experienced dose reduction or discontinuation had a similar outcome with recurrent patients (higher ORR, but indistinguishable DoR and PFS), and the most common reason for dose adjustment was nasopharyngeal necrosis. Thus, we speculate that this regimen may work better for refractory recurrent patients if they could adhere to the combined treatment.

The predictive value of PD-L1 expression and the TMB score for NPC were unclear. Similar to toripalimab,^10^ our study observed a numerically higher but nonsignificant PFS in PD-L1–positive (CPS ≥ 10) patients than in PD-L1–negative patients. Unexpectedly, the high expression of PD-L1 significantly predicted a favorable DoR in this study, suggesting that the expression of PD-L1 may predict the efficacy of this regimen if the sample size is expanded, and the CPS threshold is critical when exploring its predictive capacity for efficacy. For TMB analysis, consistent with previous reports, no significant difference in outcomes was observed between the two groups.^10^ This could be explained by the lack of DNA samples in this study and the modest relationship between the accumulation of gene mutations and the development of NPC.^48^

In addition to these notable findings, there are several limitations to consider. First, this was a single-arm study without control group, and the synergistic effect of apatinib and camrelizumab needs further validation. We are now undertaking a prospective small-sample trial to compare the treatment effects of mono-chemotherapy, chemoimmunotherapy, antiangio-immunotherapy, and antiangiochemoimmunotherapy (ClinicalTrials.gov identifier: NCT05549466). Second, the small sample size reduced the certainty of the effects observed, and further phase III randomized controlled trials are warranted.

In conclusion, camrelizumab plus apatinib showed encouraging efficacy in patients with RM-NPC. Nasopharyngeal necrosis, associated with a history of reirradiation and local recurrence, should be closely monitored and managed during treatment. Future randomized controlled trials are needed to confirm these findings.

## Data Availability

Our raw database will be deposited on the Research Data Deposit public platform (www.researchdata.org.cn). If a researcher wants to use our raw data for scientific research purposes, she or he could apply for use with our corresponding author and database administrator.
